# Endovascular treatment of pediatric ruptured intracranial dissecting aneurysm: a case report and literature review

**DOI:** 10.3389/fsurg.2025.1704284

**Published:** 2026-01-21

**Authors:** Haitong Xu, Yongkai Qin, Liyang Zhang, Jiahong Chen, Bo Li, Junfei Han, Zhengwei Huang, Yingchao Jing

**Affiliations:** Department of Neurosurgery, Huizhou Third People’s Hospital, Guangzhou Medical University, Huizhou, China

**Keywords:** communicating segment, digital subtraction angiography, endovascular interventional therapy, intracranial aneurysms, intracranial dissecting aneurysm, subarachnoid hemorrhage, whole-exome sequencing

## Abstract

Intracranial aneurysms (IAs) are uncommon in children, with an incidence of 1%–5%. However, intracranial dissecting aneurysms (IDA) account for a higher proportion (20%–50%) of all aneurysms in this age group. Pediatric IDAs typically result from vascular wall injury, potentially associated with genetic predisposition, congenital defects, or trauma. These lesions most commonly present with ischemic stroke, while subarachnoid hemorrhage (SAH) is relatively rare. Early symptoms include headache and vomiting, with severe cases potentially leading to neurological deficits. Digital subtraction angiography (DSA) remains the gold standard for diagnosis. Timely diagnosis and intervention are critical for improving prognosis. Treatment options include pharmacological therapy, endovascular intervention, and microsurgical repair. This report details a case of a 14-year-old male diagnosed with a dissecting aneurysm in the communicating segment of the left internal carotid artery (ICA). Emergency endovascular intervention with coil occlusion of the parent artery was performed. Short-term follow-up demonstrated favorable outcomes without new neurological deficits. The clinical characteristics of this condition are briefly reviewed in the context of this case.

## 1 Introduction

Intracranial aneurysms (IAs) are rare in pediatric patients, with an estimated incidence of 1%–5% (1). Pediatric intracranial dissecting aneurysms (IDAs) represent an uncommon vascular pathology typically occurring in children and adolescents during developmental stages. These lesions are characterized by intramural hematoma formation resulting from vascular wall injury or structural anomalies (2, 3). Although the precise etiology and pathogenesis remain incompletely understood, potential contributing factors include genetic predisposition, congenital vascular defects, trauma, and metabolic disorders (4, 5). Spontaneous IDAs account for approximately 45% of all pediatric intracranial aneurysms; most cases manifest with ischemic stroke or neurological deficits, while subarachnoid hemorrhage (SAH) occurs less frequently (6, 7). Initial symptoms—often precipitated by physical exertion or trauma—may include headache, vomiting, and dizziness; severe presentations can involve acute neurological deterioration such as facial asymmetry, limb weakness, or dysarthria (8). Definitive diagnosis relies on advanced vascular imaging, including computed tomography angiography (CTA), magnetic resonance angiography (MRA), or digital subtraction angiography (DSA). These modalities enable visualization of vascular architecture, confirmation of dissection, and assessment of collateral circulation (8, 9). Early diagnosis is critical for optimizing outcomes, as delayed intervention may lead to catastrophic complications, including stroke or seizure.

Recent advancements in imaging technology have improved IDA detection rates, though diagnostic challenges persist. Management strategies for unruptured IDAs may include surveillance and conservative therapy, particularly in asymptomatic or non-enlarging lesions. Conversely, once ruptured, IDAs carry a significantly poorer prognosis than saccular aneurysms, and are associated with high rebleeding rates that necessitate prompt intervention (6, 7, 10–13). Treatment methods for pediatric IDA include medications, microsurgery (e.g., aneurysm neck clipping, aneurysm wrapping, aneurysm artery ligation, etc.), and endovascular intervention (simple intracapsular embolization of aneurysm, balloon or stent-assisted aneurysm embolization, occlusion of aneurysm-bearing arteries, or placement of stent grafts to isolate aneurysms) (14, 15). While favorable neurological recovery may occur due to enhanced neuroplasticity and collateral perfusion in pediatric patients (16, 17), management must be tailored to individual patient characteristics. This report describes a 14-year-old male presenting with progressive headache and transient right upper limb weakness. DSA confirmed a dissecting aneurysm in the communicating segment of the left internal carotid artery (ICA). Endovascular interventional coil occlusion of the aneurysm-bearing artery was performed urgently. Short-term follow-up demonstrated excellent clinical recovery without neurological sequelae. The clinical features of pediatric IDAs are discussed in the context of this case.

## 2 Case report

### 2.1 Patient clinical information

**Figure 1:**
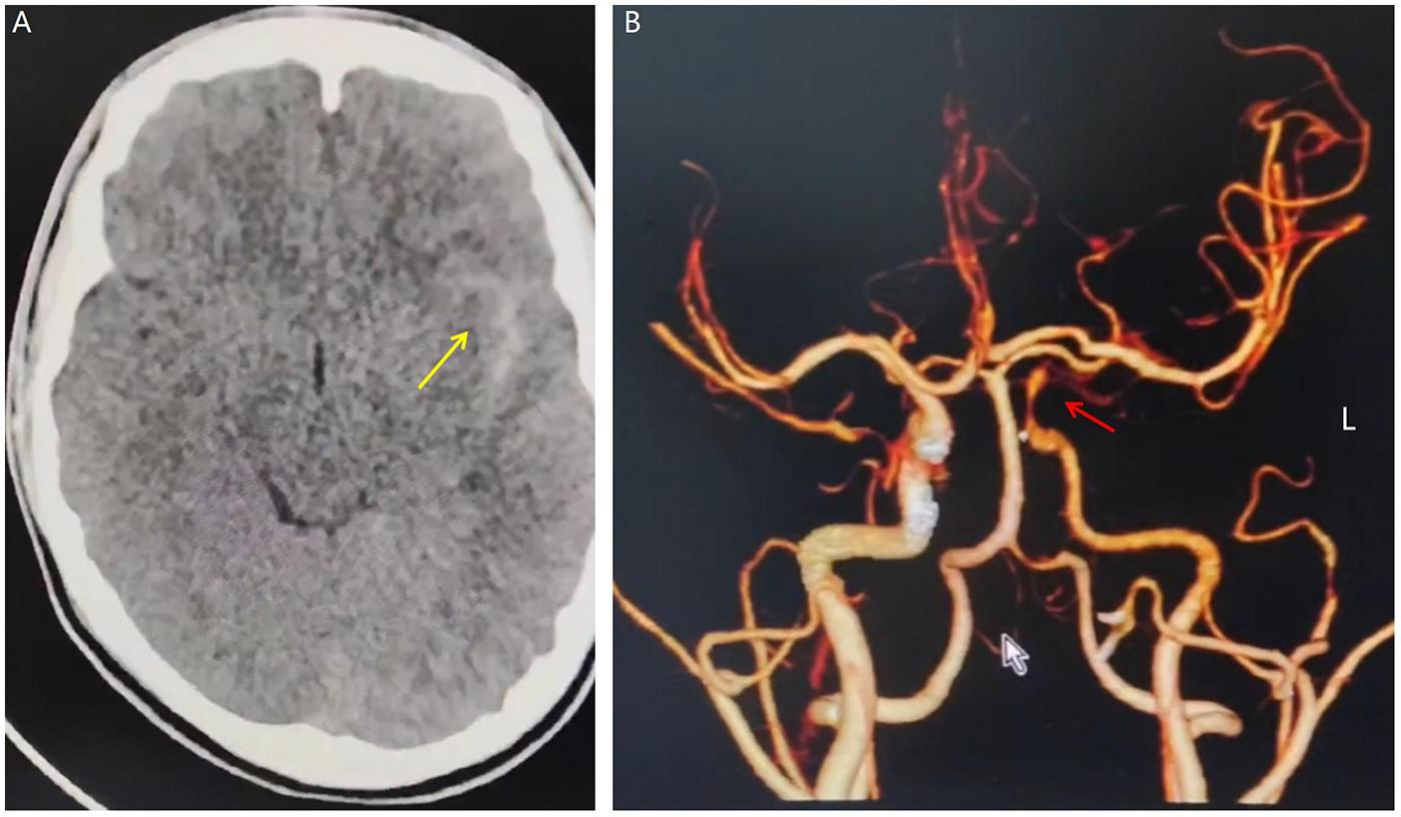
**(A)** Non-contrast cranial CT demonstrates subarachnoid hemorrhage in the left Sylvian fissure (yellow arrow). **(B)** Three-dimensional CTA reconstruction reveals segmental stenosis at the communicating segment of the left internal carotid artery (ICA), suggestive of a dissecting aneurysm (red arrow).

On June 10, 2024, a 14-year-old male presented with a 1-week history of headache and transient right upper limb weakness lasting 1 day. The persistent throbbing headache began after basketball activity (without trauma or falls), and was partially relieved by rest. No dizziness, nausea, vomiting, or limb weakness was reported initially. One day before admission, the headache intensified with transient right arm weakness. Emergency cranial CT at a local hospital revealed SAH (Figure 1A). Subsequent cranial CTA demonstrated a dissecting aneurysm in the communicating segment of the left ICA (Figure 1B), prompting immediate transfer to our institution. Physical examination on admission: Alert with mild lethargy; bilateral pupils equal and reactive (diameter 2.5 mm); mild nuchal rigidity. Glasgow Coma Scale (GCS): 14 (E3V5M6); modified Rankin Scale (mRS): grade 1; Hunt-Hess grade: grade 1; modified Fisher grade: grade 1. History included regular basketball, badminton, and chronic sleep deprivation due to gaming. Born full-term via vaginal delivery. Auxiliary tests: Chest CT and electrocardiogram were unremarkable. Complete blood count, erythrocyte sedimentation rate, vasculitis markers, and autoimmune panels showed no abnormalities.

### 2.2 Surgical procedure

**Figure 2:**
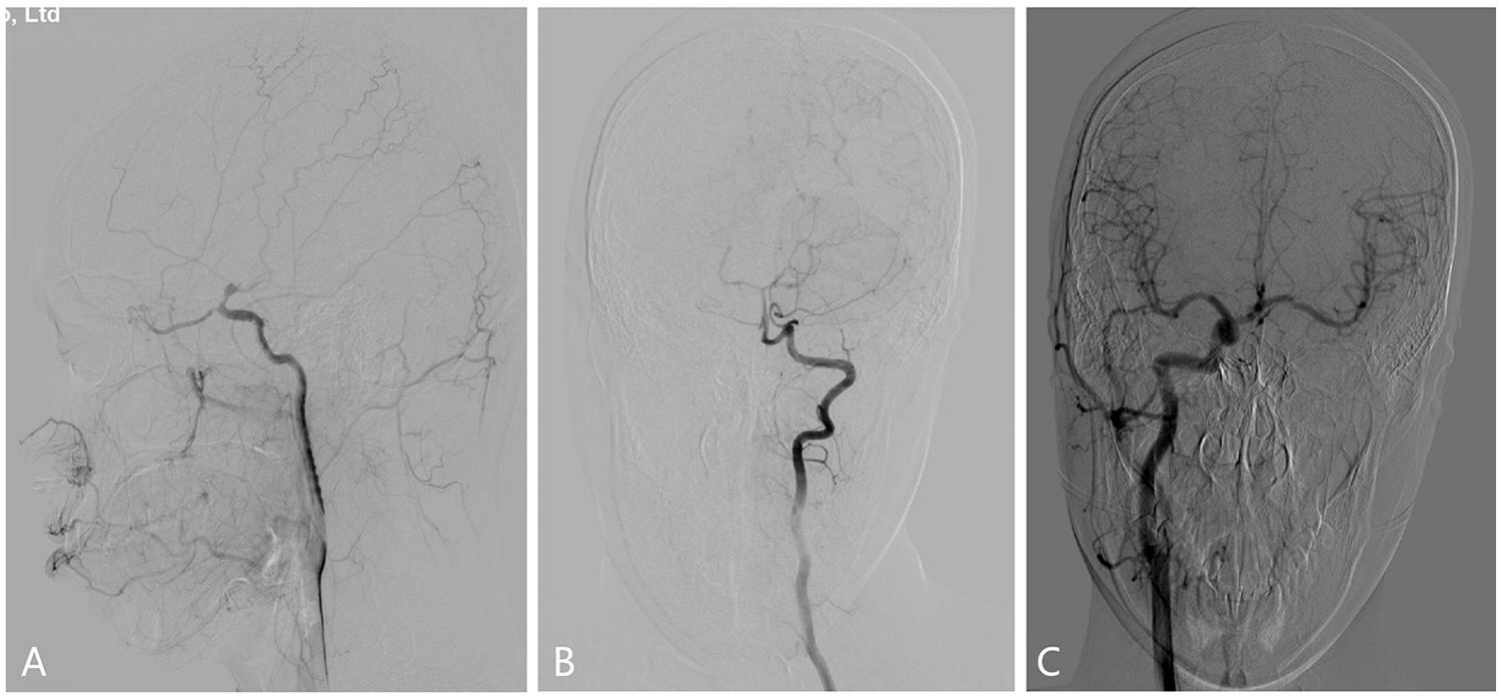
**(A)** left internal carotid arteriography showed a dissected aneurysm in the left ICA communication section, with a size of about 4.5 mm × 6.0 mm, involving the posterior communicating artery and very close to the ophthalmic artery. **(B)** Left vertebral arteriography showed good bilateral posterior cerebral arteries; **(C)** Right internal carotid arteriography showed open anterior communication and bilateral symmetrical blood flow.

**Figure 3:**
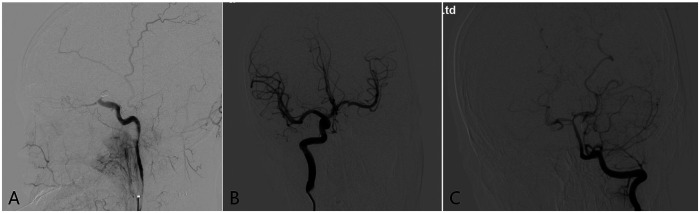
**(A)** lateral angiography of the left internal carotid artery showed that the coil was placed in the aneurysm-bearing artery to achieve occlusion of the proximal ICA communication segment, and no aneurysm filling was seen at the distal end. **(B)** Right internal carotid arteriography showed that the left anterior cerebral artery was opened through anterior communication, and blood flow compensated for the left middle cerebral artery through anterior communication, and no retrograde aneurysm filling was seen. **(C)** Left vertebral artery angiography showing that the left posterior cerebral artery fills the distal ICA artery region through the left posterior communicating artery, and no retrograde aneurysm filling is seen.

DSA was performed under emergency local anesthesia, and a dissected aneurysm was seen in the left ICA communication section with a size of about 4.5 mm × 6.0 mm, and the dissected aneurysm was relatively large, involving the posterior communicating artery, and was very close to the ophthalmic artery (Figure 2A). Left vertebral arteriography (VA) demonstrated good filling of bilateral posterior cerebral arteries (Figure 2B). Right ICA angiography confirmed that the anterior communicating artery was patent with symmetrical bilateral flow (Figure 2C). After discussion, the patient's family consented to endovascular intervention for occlusion of the aneurysm-bearing artery. The procedure was subsequently performed under general anesthesia with systemic heparinization. A catheter was positioned in the proximal C1 segment of the left ICA, and a microcatheter was advanced into the parent artery at the left ICA communicating segment. Five coils were deployed to occlude the parent artery lumen at the proximal ICA communicating segment. Post-procedural angiography confirmed successful occlusion of the target segment, with no evidence of anterograde or retrograde venous filling. The right ICA and left VA angiography showed that the left anterior cerebral artery was opened through anterior communication and the left posterior cerebral artery filled the distal ICA artery through the left posterior communicating artery (Figures 3A–C). The patient tolerated the surgery well, and there were no new neurological deficits, including decreased vision, etc. At the same time, it was shown that there was good collateral circulation around the aneurysm-carrying artery by occlusion, and blood pressure increased by 20% compared with usual throughout the perioperative period.

### 2.3 Outcomes

**Figure 4:**
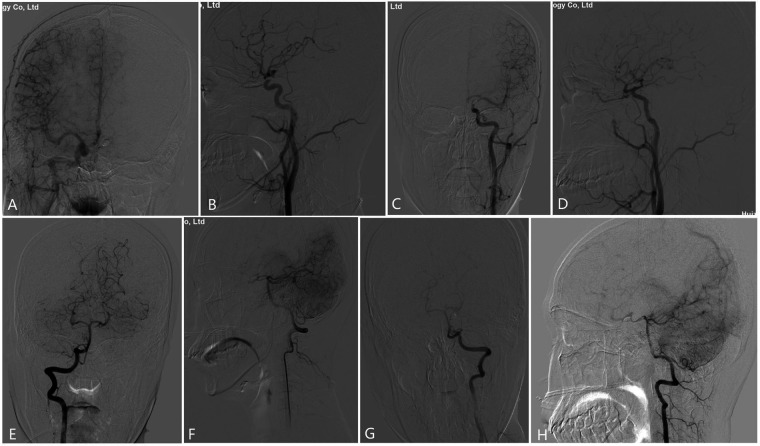
**(A,B)** anteroposterior **(A)** and lateral **(B)** angiography of the right internal carotid artery showed that the anterior circulation was open, and the blood flow compensated for the left middle cerebral artery through the anterior communication, and also regurgitated to the left ICA traffic segment. **(C,D)** left internal carotid artery anteroposterior **(C)** and lateral **(D)** angiography showed patency of the distal anterior and middle cerebral arteries, and the true lumen of the dissection was repaired and opened. **(E–H)** Bilateral (right, **E,F**; left, **G,H**) vertebral artery anteroposterior **(E,G)** and lateral **(F,H)** angiography showed bilateral posterior communicating artery blood flow open, and the communicating artery regurgitated to the communicating segment after blood flow.

On the second day of admission, the cranial CT scan showed that SAH had been basically completely absorbed, and there was no new blood or cerebral infarction. On the 3rd day of admission, the headache was relieved, no other neurological deficits were found, and he was discharged after 1 week of hospitalization with mRS grade 0. The patient returned for cerebral angiography three months post-surgery. Angiography of the right ICA revealed patency of the anterior communicating artery, which compensated for blood flow to the left middle cerebral artery and also regurgitated to the communicating segment of the left ICA. Left ICA angiography demonstrated opacification of the distal anterior and middle cerebral arteries, with no evidence of aneurysm filling, indicating gradual repair and recanalization of the true lumen of the dissection. Additionally, bilateral vertebral artery angiography showed patency of the posterior communicating artery, with blood flow returning to the communicating segment (Figures 4A–H). No recurrence of the intracranial aneurysm was observed during the follow-up period, and the true lumen of the dissection was gradually repaired and recanalized. After 13 months, there was no new-onset neurological deficit at outpatient follow-up.

## 3 Discussion

Pediatric stroke, encompassing cerebrovascular events in individuals aged 30 days to 18 years, was historically considered rare but now demonstrates increasing incidence (1.29–13.5 per 100,000 children annually), surpassing childhood brain tumors (18). While IAs are uncommon in children, ruptured IDAs represent a significant etiology of pediatric SAH, accounting for 20%–50% of all aneurysms in this age group (13). IDAs are classified as spontaneous or traumatic. Spontaneous IDAs constitute approximately 45% of pediatric aneurysms; the annual incidence of spontaneous dissecting stroke is 11.5/100,000, representing a major cause of ischemic stroke in young patients. These lesions predominantly affect the posterior circulation (e.g., basilar or vertebral arteries), manifesting as occlusive stroke or SAH (6, 8, 19). We report a rare case of SAH originating from a dissection in the communicating segment of the ICA within the anterior circulation. Such cases have been infrequently documented in recent literature.

Lasjaunias et al. demonstrated that IDA incidence increases with age but remains four times higher in children than in adults (2, 3, 20). Pediatric IDAs exhibit distinct risk profiles: trauma, infection, or congenital disorders predominate in children vs. hypertension, smoking, and alcohol abuse in adults (2–5, 8). Pathophysiology involves multifactorial mechanisms (congenital, acquired, hemodynamic), with rupture typically linked to intramural hemorrhage, causing hemorrhagic/ischemic sequelae (4, 5).

The recanalization and true lumen repair of the aneurysm-bearing artery after occlusion are not uncommon but rather a dynamic process involving multiple factors, corely associated with the physiological characteristics of pediatric blood vessels and the pathological progression of the lesion. As a key driving factor, intramural hematoma exerts a dual role: on one hand, it inhibits further separation of the intima-media of the vascular wall, combines with smooth muscle cells to synthesize fibrous tissue and form neointima, thereby repairing the vascular wall structure; on the other hand, it reduces blood perfusion in the aneurysm cavity, causes blood stasis, and creates conditions for thrombosis and aneurysm remodeling. In addition, the vascular walls of children lack an atherosclerotic basis, and endothelial cells and smooth muscle cells have stronger proliferative and reparative capacities, which can strengthen the structure through thrombus organization, lysis, and vascular wall remodeling; while the abundant collateral circulation can quickly establish retrograde perfusion, which not only avoids ischemic injury but also provides hemodynamic stimulation for repair. This characteristic highlights the fundamental difference between pediatric IDA and adult IDA: adults are difficult to achieve spontaneous repair due to degenerative changes of the vascular wall, whereas children, relying on high repair potential, the healing-driven effect of intramural hematoma, and sufficient collateral compensation, make recanalization of the aneurysm-bearing artery and repair of the true lumen a relatively common outcome (16, 17). In our case, the patient had previously been active in multiple sports and developed symptoms following strenuous exercise on the day of onset. Thus, traumatic IDA was favored over a spontaneous dissection, although the latter remained a possibility.

Diagnosing pediatric IDA remains challenging due to its rarity and heterogeneous presentations. Delayed recognition may precipitate vasospasm or stroke, underscoring the importance of neuroimaging. IDAs demonstrate characteristic angiographic features on CTA, MRA, or DSA: irregular stenosis, segmental narrowing, aneurysmal dilatation, double lumen, or occlusion (9). MRA is preferred for unruptured aneurysm surveillance given its lack of radiation (8). While CTA offers fewer artifacts for post-surgical follow-up, DSA remains essential for initial post-endovascular assessment before transitioning to MRA (8, 9). Given the risks of recurrence or *de novo* aneurysm formation, DSA-guided evaluation and potential retreatment are warranted. DSA remains the diagnostic gold standard for delineating intimal injury and collateral patterns (14–18). Advances in imaging have led to the development of IDA, which is easy to miss and misdiagnose without a clear history of trauma or other symptoms, and is extremely difficult for family members and patients to detect. In addition, there may be risk factors for genetic variants in pediatric IDA, suggesting the importance of individualized treatment (14).

Therapeutic strategies for pediatric IDA lack consensus. Common options include medications, endovascular interventions (intracapsular embolization of aneurysms alone, balloon or stent-assisted aneurysm embolization, occlusion of aneurysm-carrier arteries and blood flow diversion devices, etc.), and microsurgical procedures (e.g., aneurysm neck clipping, aneurysm wrapping, aneurysm-carrier artery ligation, etc.) (8, 14, 15). Endovascular therapy is increasingly favored for pediatric aneurysms due to favorable safety profiles and durability (7, 21–24). Prompt intervention is critical for ruptured IDAs, though robust data on postoperative stroke/rebleeding risks remain limited (22–24). While parent artery preservation is ideal, complex anatomy often precludes this goal (13–17). Coil embolization provides superior deployment accuracy over liquid embolics, reducing distal embolism risk (15). Giant or complex untreated IDAs carry poor prognoses, with high risks of hemorrhagic/ischemic stroke, particularly in children (6, 10–13, 21). Aneurysm-carrier arterial occlusion is generally well tolerated in children due to their good collateral circulation (15–17). In adolescent cases, both microsurgery and endovascular therapy have shown effectiveness, whereas in infants, bypass surgery is often not required because they are naturally able to adapt to the sacrifice of the aneurysm-bearing artery (13–17). Obviously, in our case, after the patient was found with IDA, due to his young age, after considering the risks and benefits of the patient, he obtained the consent of his family to endovascular interventional embolization of the aneurysm-carrying artery treatment plan, and immediately started the intervention. After timely treatment, the follow-up DSA showed that the collateral circulation was well compensated, and the IDA true lumen repair and aneurysm-carrying artery recanalization were found without other complications. In conclusion, optimal IDA management requires individualized strategies integrating anatomical complexity, patient age, and comorbidity profiles to maximize safety and therapeutic efficacy.

Furthermore, in recent years, a growing body of research has suggested that pediatric IDA may be associated with genetic factors. Whole-exome sequencing (WES), as an important tool, helps identify potential genetic susceptibility in affected children. Certain genetic variants related to vascular connective tissue, such as COL3A1, FBN1, SMAD3, TGFBR1/2, etc., have been reported in the literature to be potentially associated with vascular wall structural abnormalities and dissection formation (14, 25–27). For spontaneous cases without a definite history of trauma, the role of genetic factors is particularly worthy of attention. In this case, although sports-related external force is considered the primary precipitating factor, it is still recommended to combine WES in future follow-up to rule out potential hereditary vasculopathies (such as Ehlers-Danlos syndrome type IV, Loeys-Dietz syndrome, etc.) (26, 27). Incorporating genetic counseling into the individualized treatment and follow-up system helps early identify the risk of systemic vascular lesions, thereby optimizing long-term management strategies.

## 4 Conclusion

There is no consensus on treatment and follow-up regimens for pediatric IDA, so an individualized approach and lifelong follow-up are needed. Although long-term outcomes are similar, hemorrhagic cases require prolonged hospitalization, resulting in higher treatment costs; Therefore, optimizing treatment strategies is essential to improve outcomes and reduce healthcare expenditures. When choosing a treatment, a risk-benefit analysis should be used, taking into account factors such as endovascular therapy, microsurgery, or combination therapy. DSA follow-up is necessary to ensure the effectiveness of treatment. In pediatric IDA, large and complex aneurysms often require treatment to improve prognosis. Therefore, individualized treatment and lifelong follow-up are essential.

## Data Availability

The original contributions presented in the study are included in the article/Supplementary Material, further inquiries can be directed to the corresponding author.
